# Life History Trade-Offs in Tumors

**DOI:** 10.1007/s40139-018-0188-4

**Published:** 2018-11-10

**Authors:** Amy M. Boddy, Weini Huang, Athena Aktipis

**Affiliations:** 1 Department of Anthropology, University of California, Santa Barbara, HSSB 2045, Santa Barbara, CA 93106-3210 USA; 2 Complex Systems and Networks Research Group, School of Mathematical Sciences, Queen Mary University of London, Mile End Road, London, E1 4NS UK; 3 Group of Theoretical Biology, The State Key Laboratory of Biocontrol, School of Life Science, Sun Yat-sen University, Guangzhou, 510060 China; 4 Department of Psychology, Arizona Cancer Evolution Center, Biodesign Center for Biocomputation, Security and Society, Arizona State University, Tempe, AZ USA

**Keywords:** Life history trade-offs, Evolutionary theory, Ecology, Neoplastic progression, Cancer evolution

## Abstract

**Purpose:**

In this paper, we provide an overview of a life history theory and how it applies to cancer evolution.

**Recent Findings:**

We review the literature on trade-offs in tumors, focusing on the trade-offs among cellular proliferation, survival, and motility. Trade-offs are critical natural constraints for almost all evolutionary processes. Many ecological studies show that trade-offs among these cellular functions maintain a genetic diversity. In addition, these trade-offs are not fixed, but rather can shift depending on the ecological circumstances in the microenvironment. This can lead to selection for the cellular capacity to respond to these differing microenvironments in ways that promote the fitness of the cancer cell. We relate these life history trade-offs to the recently developed Evo-Eco indexes and discuss how life history theory can help refine our measures of tumor evolution and ecology.

**Summary:**

Life history theory provides a framework for understanding how the spatial and temporal variability in the tumor microenvironment—in particular resources and threats—affect trade-offs among cell survival, cell proliferation, and cell migration. We discuss how these trade-offs can potentially be leveraged in cancer therapy to increase the effectiveness of treatment.

## Introduction

Life history theory is a subtheory of the organismal evolutionary theory that seeks to explain diversity and patterns in traits such as growth, maintenance, and reproduction [1]. Life history theory posits, because resources are finite, there are trade-offs in energy allocations to these essential functions [2]. In certain environments, individuals have to allocate limited resources to multiple tasks they face. Trade-offs are critical in shaping the phenotypes that evolve since organisms typically cannot maximize all fitness-relevant traits simultaneously. These allocations have fitness consequences—organisms that allocate these scarce resources in ways that enhance their survival and reproductive success end up contributing more genes to the next generation. Just like organisms, neoplastic cells meet the necessary and sufficient conditions of natural selection: variation, inheritance, and differential reproductive success [3, 4]. Neoplastic cells with the most successful strategies will tend to be selected and maintained in the cell population.

Neoplastic cells grow in environments with ecological constraints, including resources, immune predation, and physical space, leading to life history trade-offs (see Table 1 for organismal and cellular life history comparisons). In organismal evolution, these trade-offs can depend on the resources and hazards in the environment. Life history theory provides a rich framework for how the interactions with the ecological process can influence the evolution of phenotypes. This framework can be applied to understanding the ecological and evolutionary dynamics of tumor cell populations [24]. Based on these life history principles, we may be able to predict evolutionary trajectories and help classify the phenotypic diversity found in tumors. While multiple trade-offs may exist at the cellular level, here, we will focus on the ones we think are clinically relevant for tumor cells and patient outcomes. In the next section, we highlight trade-offs in reproduction, survival, and migration.

**Table 1:** Measures of LH dynamics of a tumor. Here we draw from organismal life history (LH) theory to guide cellular LH parameters of a tumor and provide a list of potential cellular markers

Organismal LH parameters	Cellular LH parameters	Potential cellular markers
Somatic maintenance	DNA damage/repair; cell cycle control	DNA damage assays [5]; survival marker BCL-2 [6]; cell cycle arrest [7]
Reproduction	Proliferation	Ki67 [8], mitotic counts
Migration	Cell motility	Mesenchymal markers, ECM markers [9, 10]
Metabolic Rate	Glycolysis, oxidative phosphorylation	Glucose uptake and lactate production [11]
Lifespan	Telomere length, telomerase activity	Ratio of telomere length in cancer to non-cancer tissue [12]; TERT levels [13, 14]
Extrinsic mortality	Apoptosis	Tunel staining [15], caspase activity [16]
Predation	Immune markers	CD8+ T cells [17, 18], M1 macrophages [19]
Body mass	Tumor size	CT imaging
Population density	Tumor density	CT imaging
Environmental resources	Microenvironmental resources	Measure oxygen, glucose; hypoxia factors: HIF-1α, CA IX [20]
Resource distribution	Angiogenesis; necrosis	VEGF [21]; CT imaging
Somatic mutation rate	Somatic mutation rate	Multi-sample sequencing [22, 23]

*LH* life history, *ECM* extracellular matrix, *CT* computerized tomography scan

## Trade-Offs Between Reproduction and Survival

How an organism allocates resources among life history traits, such as growth, maintenance, survival, and reproduction depend on features of the organism’s ecological environment, including extrinsic mortality (e.g., predators) and availability of resources (e.g., predictable vs. unpredictable). Environments with high-extrinsic mortality and unpredictable resources tend to favor the evolution of organisms that mature early and invest in reproduction, at the cost of growth or somatic maintenance, in order to successfully reproduce during their lifetime. However, large long-lived organisms tend to occupy predictable environments with low-extrinsic mortality. In these environments, populations tend to expand until they reach the carrying capacity of the environment, and then fitness is largely determined by the ability to compete for limited resources. Thus, predictable and stable environments tend to select for organisms that invest in growth and somatic maintenance while delaying investment in reproduction [25]. Similar trade-offs may exist for cancer cells, with cells occupying unpredictable microenvironments being selected for fast replication at the expense of cell survival.

Among multicellular organisms, apoptosis and cellular proliferation are tightly linked, where high rates of apoptosis are correlated with higher rates of cellular proliferation [26]. This suggests that there is a trade-off in which cells that proliferate rapidly cannot survive very well. Tumors are made up of cells with varying rates of cellular death and proliferation [27], and this variation can provide the raw material for natural selection. Evolution of life history traits is constrained by genetic variation on which selection can act to respond to the environment. Additionally, environmental heterogeneity can select for different phenotypes and so maintain genetic variance in a population. Higher proliferation may lead to higher rates of somatic mutation. When a cell dies, it releases the space it was occupying. Whichever cell expands into this new niche first has an evolutionary advantage, a process that can promote the expansion of proliferative clones [28]. Additionally, the rate of cell proliferation and cell survival may provide insight into classifying and predicting the evolutionary potential of a tumor. For example, apoptotic rates in tumors are highly correlated with proliferation rates in breast cancer patients—suggesting a fundamental trade-off between proliferation and cell survival—and highly proliferative tumors were associated with more aggressive breast cancers [29]. In addition, in some carcinomas (review in [28]), such as colorectal and breast, pro-survival markers (BCL-2) in the cells are correlated with a good prognosis of the patient. In other words, lower cell proliferation and higher cell survival seem to be associated with better clinical outcomes. These results suggest cellular proliferation and survival, especially in terms of life history trade-offs, can be an important indicator of tumor evolvability/progression.

Indeed, markers of cell proliferation—such as Ki67 and mitotic indices—have been used in clinical settings to predict clinical outcomes. But the predictive and prognostic value of these markers is still not clear for many cancers such as breast cancer [30]. One possibility that emerges from using a life history framework is that it may not be cell proliferation per se that contributes to poorer outcomes for patients, but rather the escape from the usual trade-offs between proliferation and survival. We discuss this possibility—that cancer cells may evolve to have weaker trade-offs between proliferation and survival (as well as other traits that usually a trade-off with one another) in the later section on the strength of trade-offs. Measuring markers of proliferation and survival (see Table 1) and then combining these markers to discover tumors that have started evolving to escape the usual life history trade-offs may provide additional predictive abilities on patient outcomes.

## Trade-Offs Between Survival and Migration

Migration and/or dispersal is when an organism moves from its natural habitat to an unknown/novel environment. Migration is often costly as it requires resources and energy to move from one environment to another. Different environmental conditions influence the cost/benefit ratio of migration, including density-dependence, food resources, and predators [31–33]. These conditions may influence a broad range of migratory behaviors. An organism may accept the risk of mortality due to high predation if the resources in the area are high. Alternatively, an organism may avoid resource opportunities in regions with high-predation risk [34]. There is a life history trade-off between migration and survival. While organisms may migrate due to fluctuations in resource and environmental adversity, the benefit of finding new resources may have costs, including increased risk of predation, and hazards associated with exposure to the new environment (mismatch) [35].

Using this framework, we can make predictions based on the ecology of the tumor, for when neoplastic cells transit to a metastatic phenotype. Cellular migration, i.e., metastasis, is often lethal for patients. Tumor metastasis is responsible for 90% of cancer-related deaths [36]. From the first principles of life history theory, we can predict that metastasis is also a costly transition for neoplastic cells and subject to trade-offs [33]. Tumor cells of mesenchymal phenotype exhibit higher rates of aerobic glycolysis [37], suggesting that the motile phenotype is more energetically costly than a sessile one. For progression to metastasis, tumor cells have to transform into a dispersal phenotype (transitioning from an epithelial cell to a mesenchymal cell) [38•], and undergo many different challenges from entering the bloodstream to colonizing a new tissue, making the likelihood of cell surviving after leaving the primary tumor very low (as reviewed by [36]).

Changes in environmental conditions, such as seasons and temperature, can prompt migratory behavior in organismal evolution. In tumors, ecological variables that may influence migratory behavior at the cellular level include resources, such as oxygen and glucose, and changing the pH of the microenvironment (e.g., a “cancer swamp” [39, 40]. Measuring markers of neoplastic cell resources could be an important predictor of tumor metastasis. For example, some studies show a relationship between GLUT1 and poor patient outcomes in malignant tumors [41, 42]. However, it is important to note that different tumors may be utilizing different resources and there may be intratumor heterogeneity of resource distribution within a single tumor making predictions on a single marker for resource availability difficult to assess (as discussed in [43••]).

## Trade-Offs Between Proliferation and Migration

At the organismal level, migration helps organisms to avoid regions with high population densities where there may be more competition over resources (including kin competition) and to escape from predators and other natural enemies. There is generally a strong selection to avoid competing with kin [44]. As discussed in previous sections, both reproduction and migration are costly, trading off with survival. During organismal migration, there is a high risk of mortality, but also a high risk of not finding a suitable habitat in order to reproduce. As such, there are reproductive trade-offs with migration (see examples in insects [45–47] and plants [48, 49]). There is support for this life history trade-off at the cellular level as well. In vivo work in the model organism, *Caenorhabditis elegans* (*C. elegans*), demonstrates cellular invasion and proliferation which are distinct cellular states in which the cell must be post-mitotic to invade [50]. Applying these principles to neoplastic cells, we may predict a trade-off between metastasis and cellular proliferation. Indeed, a trade-off between proliferation and migration has been observed in cancer cells. Life history trade-offs fit well within the established “grow or go” framework [38•, 51]. This framework posits that the epithelial phenotype is a stationary (grow) phenotype and the mesenchymal phenotype is capable of movement (go) [40]. Transitioning from the go phenotype means a reduction in the grow phenotype. Even cells with a predominantly motile phenotype, such as lymphocytes, show a temporary stall in migration to proliferate [52]. Additionally, there is evidence that glioma cells with invasive phenotypes decrease their proliferation rates (reviewed in [51]) and neoplastic proliferation and invasion were shown to activate different signaling pathways in human glioblastoma cells [53].

In organismal migration, inter-individual variation is an important measure of the cost/benefits of dispersal. Within a population, individual traits may promote a broad range of migration phenotypes [35], which can be similar in tumor metastasis. Tumors that are heterogeneous may have more metastatic potential due to the competition over resources. There is recent support for heterogeneity to predict metastatic potential in the colon [54] and breast cancers [55], providing additional support that the measure of intratumor heterogeneity can be useful to predict tumor outcomes (Table 1) [22, 56, 57]. Additionally, interactions with the microenvironment play a role in the “go vs grow” phenotype exhibited by neoplastic cells, and there is support for oxygen, glucose, as well as growth factors to stimulate the tumor cells to divide. Computational modeling of dispersal has shown that spatial and temporal resource heterogeneity [32] and high rates of resource use [31] select for cell migration within a neoplasm. Other environmental components, such as tumor hypoxia, have also been associated with increased risk of metastasis [58].

## The Strength of the Trade-Offs among Cell Proliferation, Survival, and Migration Can Vary

**Fig. 1:**
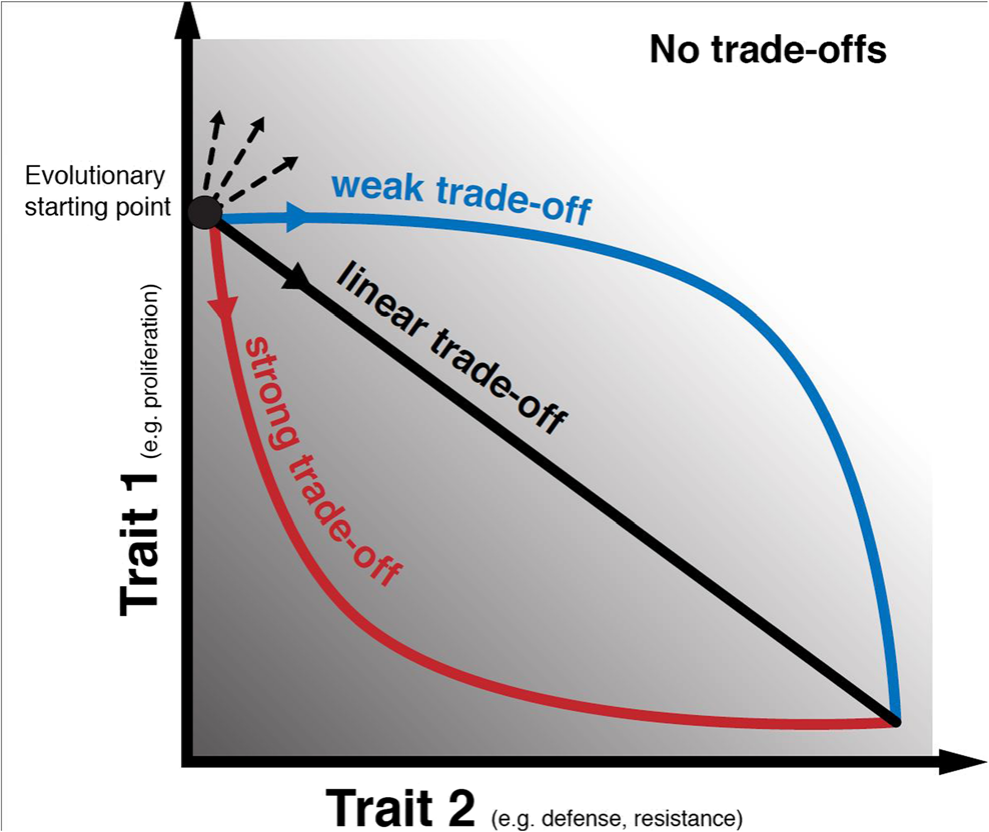
Dynamical trade-offs between two traits of cells under evolutionary processes. When two traits of tumor cells, e.g., growth (*y*-axis) and resistance to drug or ability to escape immune cell attack (*x*-axis) have a trade-off, the tumor cells evolve inside the gray area in the trait space. The improvement of resistance (trait 2) will lead to a cost of growth (trait 1). The shape of the trade-offs can be classified as linear, concave (initially weak and cheap trade-off), or convex (initially strong and costly trade-off) compared to the linear shape. It is critical for the diversity level of a tumor population. Tumor cells do not necessarily evolve along a trade-off curve with one specific shape. Instead, it is a dynamical process. For example, if the immune cells improve their ability to attack the tumor cells, the cost of the tumor cells to escape immune cells can increase over time, i.e., the tumor cells jump from an initially weaker (cheaper) trade-off curve to a stronger (more costly) one. This kind of dynamical trade-offs has been observed in bacteria populations when the bacteria cells evolve to escape the predation of ciliates

Life history trade-offs can be a driving force of the phenotype/genotype diversity in natural populations [59]. While genetic mutations or epigenetic mechanisms may be the source of various phenotypes which invest energy or resources among traits differently, the intensity of trade-offs can determine the competition level and possible coexistence of those phenotypes [60•, 61]. The strength of a trade-off can range from strong to weak and is affected by the ecological conditions, especially the availability of resources when the trade-off is due to energy allocation. For example, in one in vitro study where there was a trade-off between proliferation and survival in resistance cancer cell lines only in low glucose media conditions [62]. Additionally, in some conditions, survival can be relatively cheap, and thus, would have a weak trade-off with reproduction and/or migration. For example, in a predator-prey system, when the growth-defense trade-off is strong, extreme prey types are more likely to coexist, i.e., either the prey invests mostly in growth or in defense (Fig. 1). However, when the intensity of the trade-offs is weak (i.e., high resources may make trade-offs negligible), intermediate types can coexist with each other. Moreover, under weak trade-offs, it takes longer for the system to evolve to an equilibrium of intermediate types. Given that biological systems are inherently stochastic, we may expect a higher number of intermediate types (e.g., with intermediate reproduction and survival levels) when trade-off intensities are weak.

## Trade-Offs Can Change over Space and Time

The intensity of a life history trade-off plays a crucial role in the maintenance of diversity and ecological dynamics, but it can also be an evolving property itself. Recently, Huang et al. demonstrated in a microbial system that the growth-defense trade-off in prey shifted from a more concave shape where defense is relatively cheap—and trade-off intensity is initially weak—to be more convex with a stronger trade-off in growth-defense [60•]. These dynamical trade-offs are a natural consequence of species coevolution and it might be common in many biological systems. In other words, trade-offs exist across the tree of life, but ecological dynamics dictate the strength and intensity of the life history trade-off (Fig. 1).

Organisms can evolve to make effective life history trade-offs in “real time” as well. Behavioral flexibility or phenotypic plasticity is an effective solution when environments vary in space and/or time. Conditional life history strategies permit the organism to invest in those costly traits that will provide the greatest benefit in the current environment. Examples in the organismal literature include polyphenic insects, aphids, and crickets, where wing growth in these species is either migratory (long-winged) or reproductive (short-winged). High population densities and low food availability trigger a higher ratio of the long-winged individual that takes flight and migrates to new habitats [63]. Thus, the shape (i.e., intensity) of a trade-off should not be considered to be static but rather is a constantly changing system over space and time. Within a tumor population, cells may evolve phenotypic plasticity as well, gaining the ability to dynamically respond to the environment. These neoplastic trade-off intensities may vary in different niches of a tumor population, such as edge vs. core [64, 65•] or in resource abundant niches (near blood vessels) vs resource depleted (i.e., necrotic regions) [66, 67•].

## Applications of Life History Theory to the Evo-Eco Index

The Evo-Eco index is a new classification system to categorize the principles evolutionary and ecological dynamics of a neoplasm (see [43••]). The currently proposed evolutionary index is composed of two factors, diversity and change over time, while the ecological index measures hazards and resources. Life history theory can help us understand the importance of hazards and resources in shaping the evolutionary dynamics of neoplasms. In particular, life history theory addresses how the spatial and temporal variability in resources and threats affect these trade-offs and how these trade-offs can dynamically change. Our focus on trade-offs among proliferation, survival, and migration may provide additional insight into one of the most critical transitions in tumor biology—metastasis. Life history theory predicts that tumors with growth constraints, measured by high population densities, with patchy resource distributions, including regions with low-food resources (see Table 1 for measures) will have a higher likelihood of metastasizing. Predicting the likelihood of metastasis can be a useful clinical variable, for example in determining which liver patients are good candidates for transplant [68]. Additionally, principles from ecology can help guide clinical observations. For example, once a metastasis reaches a new tissue with lower population densities and normalized distribution of resources, it may facultatively switch to a growth and/or survival phenotype. This could potentially explain why some tumors primarily grow larger, while others disseminate and create a population of many small metastases. Understanding these underlying ecological principles can guide better predictions, and because these dynamics change over space and time—it highlights the importance of monitoring the Evo-Eco indexes longitudinally with patients.

Another application to life history trade-offs is leveraging these principles in cancer therapy to increase the effectiveness of treatment. These ecological principles can provide new insights into pharmaceutical treatments to normalize the tumor microenvironment and reduce the risk of tumor growth and/or metastasis (e.g., as is often done in adaptive therapy [69••, 70]). We know that cues from the ecology facilitate these trade-offs and manipulation of the ecology (via interventions) could prevent cellular migration or rapid proliferation and instead select for survival phenotypes and slow-growing cancers. By utilizing this trade-offs framework, we can posit that enhanced cell survival may trade-off with proliferation—and so higher cell survival may be associated with slower proliferation rates. Lowering cell proliferation rate would decrease the effective mutation rate (influencing the diversity and change over time—the Evo index [43••]). The effective mutation rate is the product of somatic mutations per cell division and the number of cell divisions per unit time and so, a higher proliferation rate should lead to a higher effective mutation rate.

Lastly, the shape of the trade-offs should be considered when we apply these trade-off concepts in neoplastic evolution. The understanding of how trade-off shapes determine that population diversity can be important for resistance diversity in tumors. For example, for tumors that are constrained by strong trade-offs, we could predict therapeutic resistance could potentially be costly for the cells in the tumor, suggesting that therapies like erzatzdroges (which “exhaust” resistant cells through flooding the environment with “fake drugs” that resistant cells pump out [71]) could be a viable approach for controlling these tumors.

## Conclusion

Here, we apply an organismal life history theory and trade-offs to neoplastic cell progression and show how this framework can be useful in understanding progression, predicting outcomes, and developing new therapeutic approaches. In this review, we focus on three fundamental trade-offs, survival vs. proliferation, proliferation vs. migration, and migration vs. survival and provide support from the literature that these trade-offs can be among cancer cells during tumor evolution. We discuss how the ecological dynamics and microenvironment of a population of cancer cells can influence the shape of these trade-offs. Additionally, we suggest cellular markers to include in future studies to provide more precise measurements of evolutionary and ecological dynamics of tumor populations. Lastly, we suggest life history theory can guide therapeutic applications for driving the evolution of a tumor population.
